# Editorial: The role of glial cells in neurodegeneration

**DOI:** 10.3389/fmmed.2023.1337286

**Published:** 2024-01-04

**Authors:** Piera Pasinelli, Kathrin Meyer, Laura Ferraiuolo, Robert A. Culibrk, Rita Sattler

**Affiliations:** ^1^ Department of Neuroscience, Weinberg ALS Center, Vickie and Jack Farber Institue for Neuroscience, Thomas Jefferson University and Jefferson Health, Philadelphia, PA, United States; ^2^ Nationwide Childrens Hospital, Abigail Wexner Research Institute, Columbus, OH, United States; ^3^ Department of Pediatrics, The Ohio State University, Columbus, OH, United States; ^4^ Sheffield Institute for Translational Neuroscience (SITraN), University of Sheffield, Sheffield, United Kingdom; ^5^ Department of Translational Neuroscience and Department of Neurology, Barrow Neurological Institute, Phoenix, AZ, United States

**Keywords:** glial, neurodegeneration, amyotrophic lateral sclerosis, frontotemporal dementia, white matter, astrocytes, microglia, oligodendrocytes

The brain is composed of distinct neuronal and glial cell-types that require sophisticated interactions and co-regulation to maintain healthy brain function. The cell-type specific complexity and heterogeneity are even more appreciated under disease conditions when minor and/or local disruptions are thought to contribute to severe dysfunction and subsequent degeneration of brain cells. Much of the knowledge in neurodegenerative disease pathogenesis thus far has come through the study of neuronal disease mechanisms without considering the glial cell component. Thus, despite the large evidence of the active involvement of glial cells in neurodegeneration, little is known about their specific contribution to disease development and progression. A comprehensive understanding of how glial cells are regulated and how they contribute to neuronal cell death will indisputably provide novel insights into mechanisms of disease initiation and progression, and consequently present new therapeutic strategies to treat and/or prevent neurodegenerative disorders.

**FIGURE 1:**
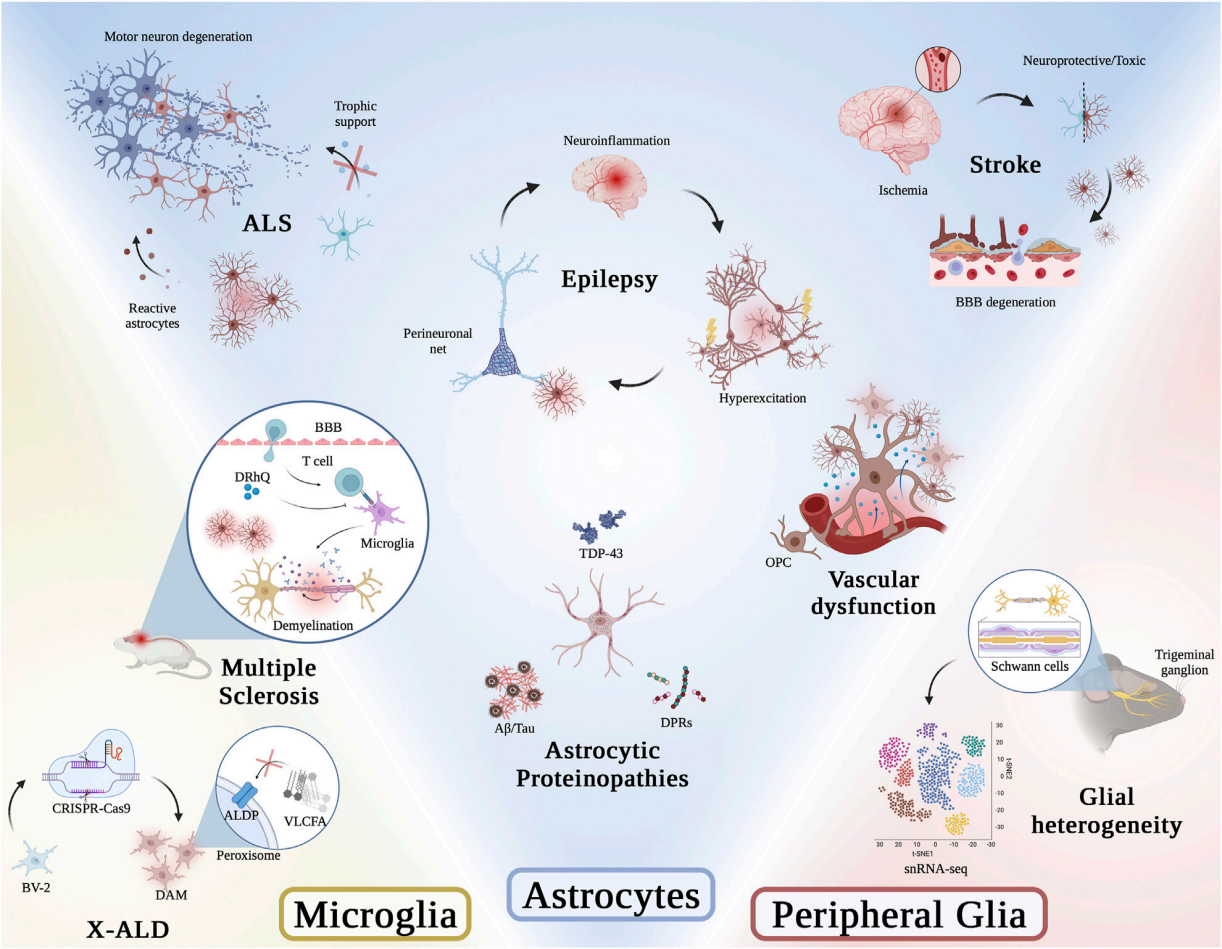
Graphical abstract of articles presented in this Research Topic. Illustration was generated using BioRender.

The current Research Topic on “The Role of Glial Cells in Neurodegeneration” in Frontiers in Molecular Medicine is aimed at offering select tidbits on the existing knowledge of glial involvement in neurodegenerative diseases. A series of 8 articles in this Research Topic are included and introduced below (see also Figure 1).

The review article by Stoklund Dittlau and Van Den Bosch provides a detailed overview on the role of astrocytes in Amyotrophic Lateral Sclerosis (ALS). While most emphasis in studying this disease has been given to the dying motor neurons, it is now widely accepted that astrocytes contribute significantly to motor neuron cell death and therefore represent a novel target for therapeutic intervention. The authors summarize the currently proposed mechanisms of astrocyte-associated toxic gain of function and loss of supportive function during motor neuron degeneration and speculate on how this knowledge might be used towards therapeutic development for people affected by ALS.

Woo and Sontheimer discuss latest findings on how astrocytes’ interactions with perineuronal nets (PNNs), components of the extracellular matrix (ECM), regulate and thereby contribute to disease progression in epilepsy. The authors highlight that there is an interplay between astrocytes and the ECM, which in turn affects neuroinflammation and subsequently neuronal function. A thorough summary provides insights into the changes in synaptic placement, ionic buffering and biophysical cellular properties caused by astrocyte-associated neuroinflammation contributing to susceptibility to or increase in seizures in epilepsy.

The review article by Collyer and Blanco-Suarez addresses the dual role (protective and/toxic) of astrocytes in the different disease phases of stroke and discusses the opportunity to target astrocytes for therapeutic intervention for stroke patients. The injury progression after an ischemic stroke is often divided into three phases: acute, sub-acute and chronic. The authors carefully examine and summarize existing literature ascribing astrocytes varying roles and contributions towards either facilitating neurodegeneration or providing neuroprotection.

Most neurodegenerative diseases are characterized by proteinopathies, which can be generally described as abnormal structural changes and subcellular localizations of specific proteins. Most of these proteinopathies have been carefully described in neurons, and Bustos and Sattler provide a thorough review summarizing the current knowledge of the presence and potential consequences of proteinopathies found in astrocytes. The authors’ report covers a broad spectrum of neurodegenerative diseases, including ALS, Frontotemporal dementia (FTD), Alzheimer’s disease (AD), Huntington’s disease (HD) and Parkinson’s disease (PD) and discusses the role of disease-specific proteinopathies in astrocyte dysfunction and how this might contribute to neuronal degeneration.

Zerimech et al. show protective efficacy of a novel third generation major histocompatibility complex Class II construct, DRhQ, towards white matter function in a mouse model for multiple sclerosis (MS). The authors showed that treatment of experimental autoimmune encephalomyelitis (EAE) mice not only altered axon excitability and delayed axon conduction, but also drastically inhibited microglial and astrocyte activation, reducing subsequent vulnerability to ischemic injury in this mouse model.

Blood brain barrier (BBB) dysfunction is a pathology common to many neurodegenerative diseases. In a comprehensive review, Gao et al. summarize the role of glial cells—astrocytes, microglia and oligodendrocytes—in regulating vascular function in the central nervous system. They then discuss how co-regulation between glial cells themselves and/or glial cells and blood vessels might go awry in neurodegenerative diseases and consequently contribute to disease pathogenesis.

The research article by Raas et al. provides evidence for microglial dysfunction in X-linked adrenoleukodystrophy (X-ALD). Performing a thorough transcriptomic analysis using a BV-2 microglial cell model of X-ALD, the authors confirmed previously described changes in very long-chain fatty acid (VLCFA) metabolism and revealed the presence of a disease-associated microglial (DAM) signature in this model system. Selected differentially expressed genes were confirmed at the protein level, strongly suggesting that microglia significantly contribute to X-ALD disease pathogenesis, and likely other peroxisomal leukodystrophies.

To better understand the heterogeneity of satellite glial cells (SGCs) in the trigeminal ganglion (TG), Chu et al. performed single cell RNA sequencing (scRNA-seq) on wild type mice. The analysis revealed similar cell clusters when compared to other peripheral sensory ganglions, including three peripheral glial cell clusters: SGSs, myeloid Schwann cells (mSCs), non-myeloid Schwann cells (nmSCs). Within the SGS cluster, cluster-specific subtypes were identified based on genes associated with extracellular matrix organization, immediate early genes, interferon beta, and cell adhesion molecules. This comprehensive data set will provide a novel reference not only for studies on physiological function in the ganglion, but also pathological conditions including nerve injuries and tumor formation.

In summary, the articles in this Research Topic represent a diverse Research Topic of recent findings supporting the important role of glial cells in the pathogenesis of neurodegenerative diseases. We strongly believe that this Research Topic will be a valuable reference for the readers and will provoke future studies in this field, advancing our understanding of the diverse roles of glial cells in the nervous system and testing new hypotheses of glial dysfunction. Our hope is that these studies will lead to the discovery and development of novel therapeutic targets and interventions, respectively.

We would like to thank the contributing authors and reviewers for their valuable expertise, time, and effort that allowed us to put together this Research Topic.

